# Prenatal Diagnosis of Peters-Plus Syndrome: A Case Report

**DOI:** 10.3390/life16010092

**Published:** 2026-01-08

**Authors:** Marina Fortún Agud, Susana Monís Rodríguez, Isidoro Narbona Arias, José Ramón Andérica Herrero, Cristina Gómez Muñoz, Marta Blasco Alonso, Jesús S. Jiménez López

**Affiliations:** 1 Obstetrics and Gynecology Department, Hospital Materno-Infantil, Hospital Regional Universitario Malaga, Avenida Arroyo de los Angeles S/N, 29011 Malaga, Spain; dr.narbona@gmail.com (I.N.A.); jranderica@hotmail.com (J.R.A.H.); kristmu@gmail.com (C.G.M.); martablascoalonso@gmail.com (M.B.A.); jesuss.jimenez@uma.es (J.S.J.L.); 2 Research Group in Maternal-Foetal Medicine Epigenetics Women’s Diseases and Reproductive Health, Biomedical Research Institute of Malaga (IBIMA), 29010 Malaga, Spain; 3 Department of Surgical Specialties, Biochemistry and Immunology, University of Malaga, 29010 Malaga, Spain

**Keywords:** Peters-plus syndrome, Peters anomaly, anterior chamber reye defects, *B3GLCT* gene, brachydactyly, corneal opacity

## Abstract

Peters-Plus syndrome is a rare autosomal recessive disorder caused by biallelic pathogenic variants in the B3GLCT gene and characterized by multisystem involvement. Fewer than 100 cases have been reported to date, and only a limited number have been diagnosed prenatally. Prenatal identification is challenging due to the variable and non-specific nature of fetal findings and the frequent absence of detectable ocular anomalies during routine ultrasound. We report a prenatal diagnosis of Peters-Plus syndrome in a monochorionic diamniotic twin pregnancy, based on the progressive identification of early-onset intrauterine growth restriction, rhizomelic limb shortening, craniofacial dysmorphism, and mild central nervous system abnormalities. Standard cytogenetic and chromosomal microarray analyses were normal, prompting extended genetic testing. Prenatal exome sequencing identified a homozygous pathogenic splice-site variant (c.660+1G>A) in *B3GLCT* in both fetuses, confirming the diagnosis. This case highlights the importance of recognizing suggestive multisystem prenatal findings and the crucial role of advanced genetic testing in achieving an accurate prenatal diagnosis. Early molecular confirmation enables appropriate parental counseling regarding prognosis, recurrence risk, and future reproductive options.

## 1. Introduction

Peters-Plus syndrome is a rare polymalformative condition of unknown current prevalence, with equal sex distribution and a high incidence in consanguineous families. Approximately 100 cases have been reported in the literature, highlighting its exceptional rarity. Peters-Plus syndrome is a rare polymalformative condition with a prevalence <1/1,000,000 and ~100 cases reported worldwide, showing equal sex distribution and high consanguinity rates. It is characterized by ocular abnormalities of the anterior chamber—most notably Peters anomaly—along with additional findings such as limb shortening, cleft lip, growth impairment, and variable intellectual disability. Following clinical suspicion, a definitive diagnosis is achieved genetically by identifying biallelic pathogenic variants in B3GLCT. This autosomal recessive disorder implies that parents of an affected child are asymptomatic carriers. Management focuses on the individual’s clinical manifestations, primarily corneal transplantation in cases of severe corneal opacification, typically performed between 3–6 months of age to prevent amblyopia [1,2,3,4,5].

Given the very limited number of prenatally diagnosed cases reported to date, this case illustrates the spectrum of prenatal ultrasound findings that should raise suspicion of Peters-Plus syndrome and highlights the role of advanced genetic testing in achieving an accurate diagnosis.

The study was approved by the Institutional Research Ethics Committee. This study was conducted in accordance with the Declaration of Helsinki and approved by the Ethics Committee of the CEI de Provincial Centre of Malaga, Spain, protocol code SICEIA-2024-002941 and date of approval 2 December 2024.

## 2. Case Description

**Figure 1:**
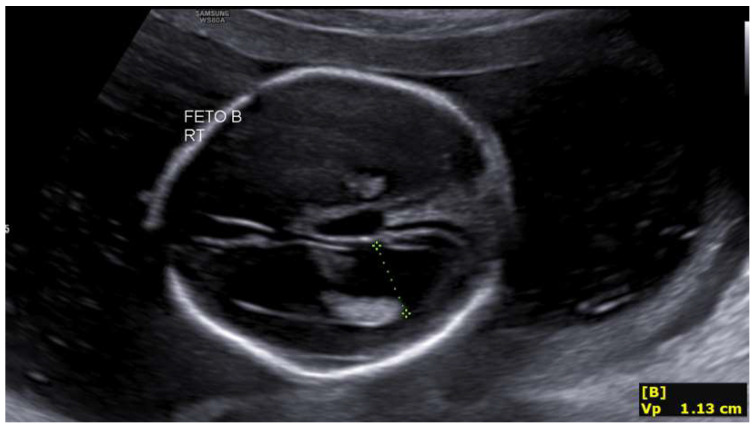
Ventriculomegaly in the second fetus at 19 + 4 weeks.

**Figure 2:**
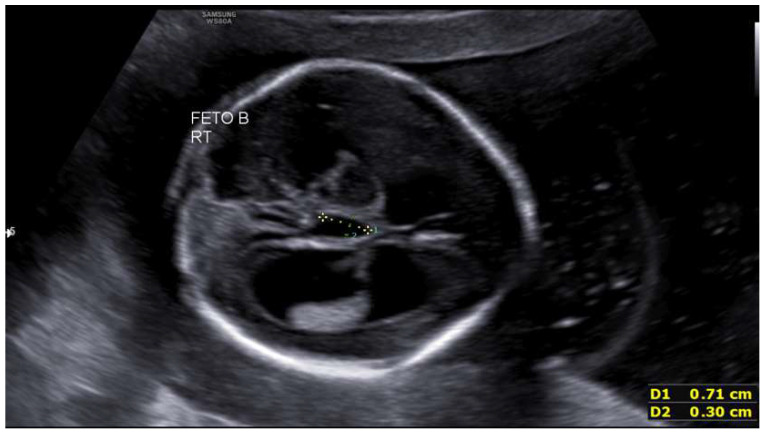
Third ventricle dilation in the second fetus at 19 + 4 weeks.

A 33-year-old primigravida with a spontaneous monochorionic diamniotic twin pregnancy was referred for routine prenatal care. The patient had no relevant personal or family medical history, and a first-trimester ultrasound showed normal fetal morphology with low-risk combined aneuploidy screening. At 19 + 4 weeks, both fetuses showed biometry lagging gestational age (fetus 1: 18 + 1 w, 235 g; fetus 2: 17 + 4 w, 209 g, single umbilical artery). Fetus 2 exhibited bilateral ventriculomegaly (anterior horns 6 mm, posterior 11–13 mm) and third ventricle dilation (7.3 mm). These measurements fall within normal limits Figure 1 and Figure 2. Intertwin weight discordance was 11%, prompting amniocentesis. 

The main findings included biometry not concordant with gestational age in both fetuses and a central nervous system malformation in the second fetus, characterized by bilateral ventriculomegaly, whereas the fetus located in the left hemiabdomen demonstrated normal lateral ventricles without fulfilling criteria for ventriculomegaly.

Based on these findings, etiological evaluation was recommended, and an amniocentesis was planned for genetic and infectious studies.

Amniocentesis was performed without complications at 20 + 2 weeks, and sequential prenatal genetic tests were requested, including QF-PCR, karyotype, microarray (CGH/SNP), and targeted clinical exome sequencing.

Initial QF-PCR analysis demonstrated a normal chromosomal complement, with no aneuploidies of chromosomes 13, 18, or 21, and male sex (XY) in both fetuses. Subsequent high-resolution microarray confirmed the absence of pathogenic or uncertain copy-number variants, with no findings to explain the ventriculomegaly. Cytomegalovirus PCR in amniotic fluid was negative in both fetuses.

Biweekly ultrasound evaluations were performed to monitor fetal development.

**Figure 3:**
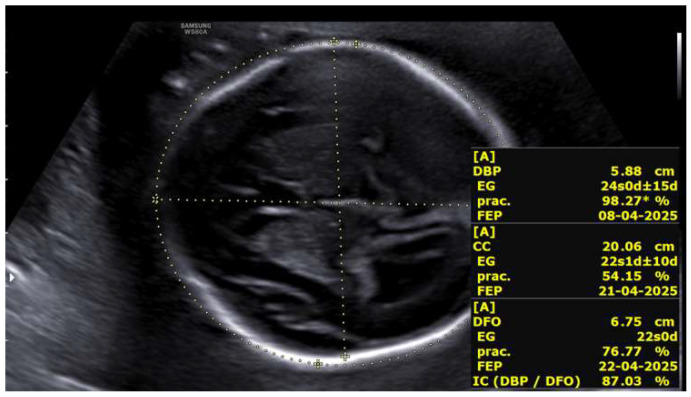
Cephalic index >85% (brachycephaly) in the fetus located in the left hemiabdomen at 23 + 5 weeks.

At 23 + 5 weeks, the fetus located in the left hemiabdomen, with positive cardiac activity and fetal movements and located in the left hemiabdomen in cephalic presentation, showed biometry consistent with 22 + 5 weeks, a three-vessel umbilical cord, and normal umbilical artery Doppler. The middle cerebral artery Doppler indicated the absence of fetal anemia. Intracranial evaluation revealed normal lateral ventricles and supratentorial structures, with a cephalic index of 87%, compatible with brachycephaly (Figure 3).

The second fetus, with positive cardiac activity and fetal movements and located in the right hemiabdomen in cephalic presentation, showed biometry consistent with 21 + 2 weeks, a single umbilical artery, and umbilical Doppler with preserved diastolic flow. The middle cerebral artery Doppler showed no evidence of anemia. Intracranially, mild bilateral ventriculomegaly was observed (anterior horns 5 mm; posterior horns 9.7–10.6 mm) along with a 5.4 × 2.8 mm interhemispheric cyst compatible with an interhemispheric cyst. The cerebral sulci and corpus callosum appeared normal. Intertwin estimated fetal weight discordance was 22%. These findings indicated a globally favorable evolution in the fetus located in the left hemiabdomen and persistent neurodevelopmental abnormalities in the second fetus. The sequential genetic testing followed SEGO/SMFEG guidelines: initial QF-PCR, karyotype, and 750K CGH/SNP microarray (turnaround < 2 weeks) ruled out common aneuploidies (13/18/21) and pathogenic CNVs, which were negative and did not explain ventriculomegaly. Clinical exome sequencing (NGS, mean coverage 100×, >95% exonic regions ≥ 20×) was deferred to 23 + 5 weeks due to phenotypic discordance in monochorionic twins, prioritizing rapid first-tier tests to minimize diagnostic uncertainty in multiple gestation while escalating to trio-exome for rare syndromes.

Given the normal prior genetic studies, expanded analysis via next-generation sequencing of the clinical exome was performed. A homozygous c.660+1G>A variant in the B3GLCT gene, located at the canonical splice site of exon 8, was identified in both fetuses. This variant is described as pathogenic in international databases and is associated with Peters-Plus syndrome.

**Figure 4:**
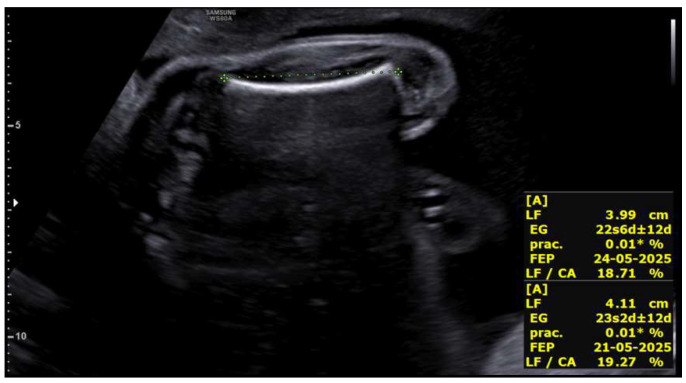
Rhizomelic shortening of the lower limb in the fetus located in the left hemiabdomenat 27 + 2 weeks.

**Figure 5:**
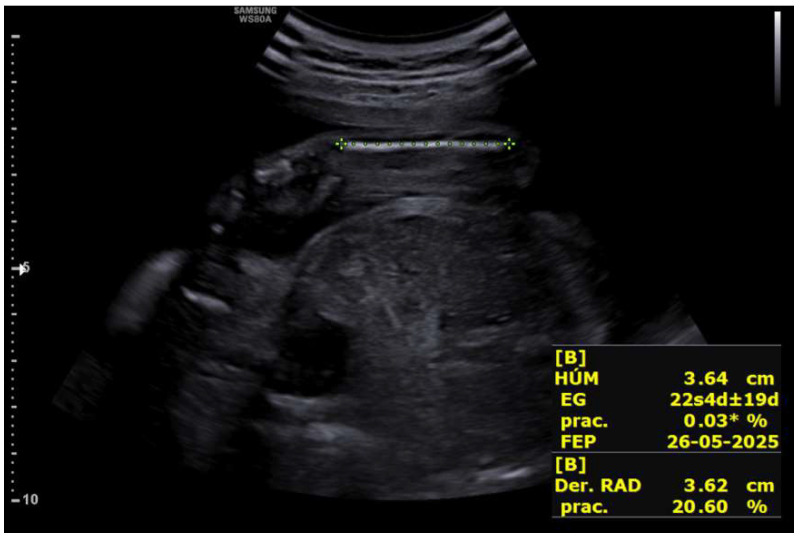
Rhizomelic shortening of the upper limb in the second fetus at 27 + 2 weeks.

Upon receiving the genetic results for both fetuses, consistent with Peters-Plus syndrome, a consultation at 27 + 2 weeks was conducted to inform the parents about the genetic nature, clinical prognosis, and functional implications of the disorder, including possible ocular, neurological, skeletal, and systemic complications, as well as future reproductive options. A detailed neurosonographic assessment of both twins was also performed, revealing stage I intrauterine growth restriction in both fetuses associated with marked rhizomelia (Figure 4 and Figure 5).

The fetus located in the left hemiabdomen showed biometry corresponding to 25 + 2 weeks with an estimated fetal weight of 833 g (3rd percentile), normal Doppler, a three-vessel umbilical cord, and a general morphological exam without major structural anomalies. Neurosonography showed lateral ventricles within normal limits, a corpus callosum of adequate morphology, and preserved cortical development. No ventriculomegaly or ocular abnormalities were identified, except for mild midfacial hypoplasia compatible with the syndrome.

**Figure 6:**
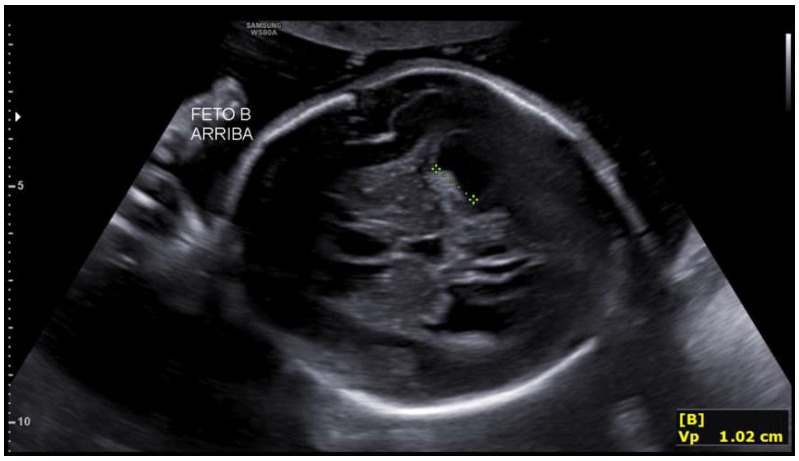
Mild bilateral ventriculomegaly in the second fetus at 27 + 2 weeks.

The second fetus showed biometry corresponding to 24 weeks with an estimated fetal weight of 686 g (0.23rd percentile), a single umbilical artery, and umbilical Doppler with preserved diastolic flow. Intracranially, mild-to-moderate bilateral ventriculomegaly was observed (Figure 6), along with third ventricle dilation, a 5 × 3 mm interhemispheric cyst, and a thin but present corpus callosum. The remaining supratentorial and posterior fossa structures showed maturation appropriate for gestational age.

Both fetuses demonstrated marked rhizomelia without additional evident skeletal anomalies. Intertwin estimated fetal weight discordance was 17.6%. Ventriculomegaly was defined per ISUOG: posterior horns > 10 mm (Fetus 2: 11–13 mm at 19 + 4w, z-score +2.5; 9.7–10.6 mm at 23 + 5 w). Rhizomelia: femur/humerus < p10 (z-score < −1.28) with proximal segment disproportion (Hadlock/Papageorghiou). Delayed sulcation on MRI (27 w, Timur-Zagorodhna score): shallow cingulate sulcus, underdeveloped Sylvian fissure. All imaging by a single expert operator (Voluson E10); multidisciplinary consensus review (2 fetal radiologists, non-blinded to clinical context).

Prenatal genetic studies (QF-PCR, 750K microarray) confirmed a normal male karyotype in both fetuses without relevant numerical or structural chromosomal abnormalities. The extended prenatal exome identified the pathogenic c.660+1G>A B3GLCT variant in both fetuses, consistent with autosomal recessive Peters-Plus syndrome. Cytomegalovirus PCR in amniotic fluid was negative. Complete TORCH screening (Toxoplasma, rubéola, herpes, syphilis, parvovirus B19) was negative by maternal serology and amniotic fluid PCR. Primigravida (33 y) had no relevant history: normal metabolic profile (glucose/HbA1c), no toxic/medication/teratogen exposures; first-trimester ultrasound normal.

The ultrasound findings—rhizomelia, mild-to-moderate brain anomalies, subtle dysmorphic features, and additional structural abnormalities—were compatible with the phenotypic spectrum of the syndrome.

Given these imaging and genetic findings, the patient expressed her desire to proceed with pregnancy termination under legal provisions for severe and incurable fetal disease. Fetal MRI was requested for anatomical characterization, parental segregation studies were initiated, and the case was submitted for evaluation by the hospital’s Clinical Committee for Pregnancy Termination.

Fetal MRI at 27 weeks identified the cavum septi pellucidi and velum interpositum in both fetuses, with normal configuration of the third and fourth ventricles, cerebellum, brainstem, and craniocervical junction. The fetus located in the left hemiabdomen, in cephalic presentation, showed mild bilateral ventriculomegaly with atrial diameter of approximately 12 mm. Cortical maturation assessment revealed delayed sulcation, with a shallow cingulate sulcus and a Sylvian fissure symmetric but underdeveloped for gestational age. Calcarine, parieto-occipital, and convexity sulci were identifiable and preserved. The second fetus, in transverse lie, showed lateral ventricles at the upper limit of normal (10–11 mm) without definitive criteria for ventriculomegaly. Partial delay in sulcation was also noted, with a unilateral shallow cingulate sulcus and less-developed convexity sulci. The Sylvian fissure, calcarine sulci, and parieto-occipital sulci appeared age-appropriate. In conclusion, MRI confirmed mild ventriculomegaly in the fetus located in the left hemiabdomen and, in both fetuses, partial and non-uniform delayed sulcation compatible with mild-to-moderate cortical developmental abnormalities.

The Clinical Committee for Pregnancy Termination, based on imaging findings, genetic results, and current literature regarding functional implications and clinical prognosis of Peters-Plus syndrome, determined that the case met criteria for legal termination due to serious and incurable fetal disease.

Fetocide was performed at an external facility without complications, and the patient was admitted to our center for completion of the termination. Cervical ripening was induced with oral mifepristone followed by vaginal prostaglandins. Fourteen hours after admission, vaginal delivery occurred: the fetus located in the left hemiabdomen weighed 1200 g (Apgar 0/0) and the second fetus weighed 990 g (Apgar 0/0). The patient had an uncomplicated postoperative course and was discharged after 24 h.

Parental segregation studies confirmed that both parents were heterozygous carriers of the variant, while both fetuses were homozygous affected, establishing a 25% recurrence risk in future pregnancies. These findings confirmed the genetic etiology of the ultrasound abnormalities, representing the first prenatal diagnosis in this family, and preimplantation genetic testing (PGT) was recommended for future reproductive planning.

In July 2025, the patient reported a new spontaneous pregnancy. An early ultrasound was scheduled to assess viability, and chorionic villus sampling was recommended given the genetic status of both parents and prior obstetric history.

At 9 weeks, ultrasound identified a viable single embryo consistent with gestational age, accompanied by a retroplacental hematoma and a small subserosal fibroid. During the first-trimester screening ultrasound at 12 + 3 weeks, major fetal malformations were identified, including an occipital meningocele with indirect signs of neural tube defect (crash sign, “dry brain,” and increased intracranial translucency), and unilateral cleft lip. Additional soft markers for aneuploidy were observed (increased nuchal translucency, tricuspid regurgitation, and single umbilical artery), whereas kidneys and limbs appeared normal.

Transcervical chorionic villus sampling was performed without complications, with subsequent confirmation of fetal viability. Rapid QF-PCR demonstrated diploid complement for chromosomes 13, 18, 21, and XY. Later molecular analysis confirmed fetal homozygosity for the c.660+1G>A variant, inherited biallelically, consistent with the expected autosomal recessive disorder.

After multidisciplinary counseling regarding the perinatal prognosis—considered severely compromised due to the malformations and molecular diagnosis—the patient opted for legal pregnancy termination, which proceeded uneventfully.

She was again counseled regarding the 25% recurrence risk in future pregnancies and referred for specialized reproductive counseling.

## 3. Discussion

Patients with Peters-Plus syndrome may present with multiple physical anomalies characteristic of this condition. The typical triad includes anterior chamber defects, short stature, and brachydactyly. Peters-Plus syndrome is a very rare autosomal recessive disorder, with fewer than 100 cases reported to date and only a limited number diagnosed prenatally. The present case contributes to the literature by illustrating the diagnostic pathway and challenges of prenatal identification of this condition, particularly in the absence of obvious ocular anomalies during mid-gestation ultrasound [1,4,6].

The most common defect affects the anterior segment of the eye and is defined as Peters anomaly, consisting of central corneal opacity associated with thinning or absence of Descemet membrane and posterior corneal thinning with iridocorneal adhesions. Two types exist: type I, a milder form involving only the cornea and often unilateral; and type II, a more severe variant with lens abnormalities and poorer visual prognosis. Common ocular complications include cataracts and glaucoma, with the latter present at birth in 50% of patients. Although Peters anomaly is the most frequent ocular defect, other findings such as mild mesenchymal dysgenesis or iris coloboma may also occur [1,3,5,6,7,8].

Skeletal alterations include growth restriction, more pronounced in height than weight, with rhizomelic limb shortening and characteristic brachydactyly. Clinodactyly of the fifth finger may be prominent. Limited elbow range of motion or hypermobility in other joints may be observed. Growth hormone deficiency is common and typically responds well to replacement therapy. Growth restriction begins prenatally, although not always detected. Adult height ranges from 1.28–1.51 m in females and 1.41–1.55 m in males. Although no specific imaging findings have been described, typical early-onset arthritis and thoracic hemivertebrae may be identified [1,2,3,6,9].

Intellectual disability occurs in 78–83% of patients and ranges from mild to severe forms; severe impairment is seen in up to 26% of affected individuals. CNS anomalies may include microcephaly, neural tube defects, and corpus callosum agenesis, although structural abnormalities may be absent even in patients with intellectual disability, indicating poor correlation between imaging findings and neurodevelopmental outcomes. Epilepsy is another possible manifestation [1,2,3,4,6].

### 3.1. Prenatal Diagnosis: Challenges and Key Ultrasound Findings

Prenatal diagnosis of Peters-Plus syndrome is inherently challenging due to the variable and non-specific nature of its fetal manifestations. Classic postnatal features such as anterior chamber defects and corneal opacity are difficult to detect prenatally, especially in the second trimester. Consequently, suspicion usually arises from indirect and multisystemic findings rather than from ocular abnormalities alone.

Review of the few reported prenatally diagnosed cases reveals a recurring pattern of findings, including early-onset intrauterine growth restriction, rhizomelic limb shortening, craniofacial anomalies (such as cleft lip and midfacial hypoplasia), and mild central nervous system abnormalities, particularly ventriculomegaly or corpus callosum anomalies. These features, summarized in Table 1, were also present in our case and guided the decision to pursue extended genetic testing.

Importantly, none of these findings is individually pathognomonic. However, their co-occurrence, particularly in the setting of otherwise normal first-tier genetic testing, should prompt consideration of rare syndromic conditions and justify the use of prenatal exome sequencing.

A summary table of prenatal and clinical findings of Peters-Plus syndrome is shown below (Table 1).

Es importante recalcar que se trata de una entidad que se presenta por medio de alteraciones variables y no específicas de dicha patología, por lo que el diagnóstico prenatal basado exclusivamente en los hallazgos ecográficos puede resultar muy complicado [5].

### 3.2. Peters Anomaly Versus Peters-Plus Syndrome

Initial suspicion arises in patients with bilateral anterior chamber anomalies, although unilateral cases occur, especially when associated with skeletal dysplasia (short limbs with broad distal segments), characteristic facial features (cleft lip/palate, exaggerated cupid’s bow, short palpebral fissures, ear anomalies), and variable intellectual disability. Family history with similar findings strengthens suspicion [1,5,6].

It is essential to distinguish Peters anomaly from Peters-Plus syndrome, as these entities are often used interchangeably but represent different clinical conditions. Peters anomaly refers to an isolated developmental defect of the anterior segment of the eye and may occur sporadically or in association with mutations in genes such as *PAX6*, *PITX2*, or *CYP1B1*. In contrast, Peters-Plus syndrome is a multisystem disorder caused by biallelic pathogenic variants in *B3GLCT* and is characterized by Peters anomaly in combination with growth restriction, skeletal anomalies, craniofacial dysmorphism, and variable neurodevelopmental impairment.

In the prenatal setting, ocular findings may be absent or subtle, making this distinction particularly difficult. In our case, the diagnosis was established not by direct visualization of anterior segment anomalies but by the recognition of a syndromic pattern and subsequent molecular confirmation [1,6,7,10].

Inheritance is autosomal recessive; thus, parents are asymptomatic heterozygous carriers. Each sibling has a 25% chance of being affected, 50% chance of being a carrier, and 25% chance of being unaffected. Carrier testing and preconception counseling are recommended, including discussion of prenatal or preimplantation genetic testing. Syndromes with similar findings but without B3GLCT mutations are classified as Peters-Plus-like [1,2,3,4,9].

### 3.3. Role of Prenatal Genetic Testing and Counseling

Management is symptom-directed and multidisciplinary. Penetrating keratoplasty is indicated for severe bilateral corneal opacification (Peters type I/II), ideally at 3–6 months to prevent amblyopia; keratoprosthesis follows graft failures. Glaucoma (50% cases) requires medical/surgical control, though outcomes are poorer (32% IOP control) than primary congenital glaucoma. Growth hormone replacement addresses prenatal-onset short stature (responsive in most); early interventions target developmental delay (78–83%), with echocardiography/renal ultrasound for systemic anomalies. Keratoprosthesis is considered after repeated graft failures. In milder cases, release of iridocorneal adhesions or medical/surgical glaucoma treatment may be performed. Congenital glaucoma associated with Peters anomaly is more difficult to treat and has poorer outcomes compared to primary congenital glaucoma; adequate intraocular pressure is achieved in only 32% of cases, and long-term visual outcomes are poor due to underlying ocular pathology [1,6,8].

This case underscores the critical role of prenatal genetic testing, particularly next-generation sequencing, in fetuses with unexplained growth restriction and multisystem anomalies. When standard cytogenetic and microarray analyses are normal, prenatal exome sequencing can provide a definitive diagnosis, enabling accurate prognostic counseling and informed parental decision-making.

Early molecular diagnosis also has significant implications for genetic counseling, allowing identification of parental carrier status and discussion of recurrence risk and reproductive options, including prenatal or preimplantation genetic testing in future pregnancies.

## 4. Conclusions

Peters-Plus syndrome is a rare autosomal recessive disorder characterized by marked phenotypic variability and multisystem involvement. The diagnosis relies on the identification of biallelic pathogenic variants in the *B3GLCT* gene, which confirms the underlying molecular defect and enables accurate genetic counseling.

The present case highlights the challenges of prenatal diagnosis of Peters-Plus syndrome, as typical ocular manifestations may not be readily detectable during routine fetal ultrasound. Instead, indirect prenatal findings, such as early-onset intrauterine growth restriction, rhizomelic limb shortening, craniofacial anomalies, and mild central nervous system abnormalities, should raise suspicion of an underlying genetic syndrome and prompt further diagnostic evaluation.

This report emphasizes the value of advanced prenatal genetic testing, particularly exome sequencing, in cases with unexplained multisystem abnormalities and normal first-tier cytogenetic studies. Molecular confirmation allows informed parental counseling regarding prognosis, recurrence risk, and available reproductive options.

Early identification of parental carrier status is essential for appropriate genetic counseling and facilitates consideration of targeted prenatal testing or preimplantation genetic testing in future pregnancies.

## Figures and Tables

**Table 1:** Main Prenatal and Clinical Findings of Peters-Plus Syndrome.

System/Region Affected	Typical Finding	Description
Fetal Growth	Intrauterine growth restriction (IUGR)	Prenatal onset in many cases; may progress during late gestation.
Craniofacial	Anterior chamber anomalies (Peters anomaly)	Corneal opacity, iridocorneal adhesions, increased anterior segment echogenicity; absence of the lens in severe cases.
	Microphthalmia/partial anophthalmia	Underdeveloped or small ocular globes.
	Micrognathia	Small mandible with retruded facial profile.
	Elongated philtrum	Characteristic feature with prominent upper lip groove.
	Cleft lip and/or palate	Detectable on 3D facial ultrasound or coronal planes.
Skeleton / Limbs	Rhizomelic shortening	Disproportionately shortened proximal limb segments.
	Brachydactyly	Short digits; broad hands and feet.
	Fifth-finger clinodactyly	Curvature of the little finger toward the fourth finger.
	Single palmar crease	Transverse palmar crease (typically identified postnatally).
Genitourinary System	Hydronephrosis	Pelvicalyceal dilatation.
	Ureteral duplication	Double ureteral system.
	Renal hypoplasia	Small or underdeveloped kidneys.
	Hypospadias	Urethral meatus located on the ventral aspect of the penis.
	Cryptorchidism	Undescended testes.
	Rudimentary uterus/vagina	Underdeveloped or absent Müllerian structures.
Cardiac	Septal defects (atrial or ventricular)	Congenital structural heart defects.
	Subvalvular aortic stenosis	Obstruction below the aortic valve.
	Hypoplastic left heart	Underdevelopment of left-sided cardiac structures.
Central Nervous System	Intellectual disability (postnatal; not detectable prenatally)	May occur with or without structural CNS abnormalities.
	Mild structural abnormalities	Partial agenesis or hypoplasia of the corpus callosum may occur.
Other Clinical Features	Short stature, coarse facial features, short neck, inguinal hernias, rectus diastasis	Multisystem involvement characteristic of the syndrome.

## Data Availability

The original contributions presented in the study are included in the article; further inquiries can be directed to the corresponding author.
